# Oral metal immunotoxicity in rodents: a recoverability-aware systematic review and within-study quantitative reanalysis of splenic SRBC-TDAR

**DOI:** 10.3389/ftox.2026.1855035

**Published:** 2026-07-22

**Authors:** Iliana C. Martínez-Ortiz, Igor Garcia-Atutxa, Carlos Machain-Williams, Miguel Ángel Reyes-López, Francisca Villanueva-Flores

**Affiliations:** 1 Centro de Investigación en Ciencia Aplicada y Tecnología Avanzada (CICATA) Unidad Morelos, Instituto Politécnico Nacional, Boulevard de la Tecnología, Xochitepec, Morelos, Mexico; 2 Escuela Politécnica Superior, Universidad Católica de Murcia (UCAM) Av. de los Jerónimos, Murcia, Spain; 3 Estudios en Una Salud, Unidad Profesional Interdisciplinaria de Ingeniería Campus Palenque, Instituto Politécnico Nacional, Palenque, Chiapas, Mexico; 4 Centro de Biotecnología Genómic, Laboratory of Conservation Medicine, Instituto Politécnico Nacional, Reynosa, Tamaulipas, Mexico

**Keywords:** antibody-forming cells, Chemical Effects in Biological Systems, immunotoxicity, oral heavy metals, recoverability, rodent models, splenic sheep red blood cell T-dependent antibody response

## Abstract

**Introduction:**

Public toxicology databases are increasingly used for evidence synthesis, but their value depends not only on the availability of studies but also on whether endpoint-level data can be recovered for reuse. Using the Chemical Effects in Biological Systems (CEBS) database as the primary source, we reviewed oral heavy-metal immunotoxicity in rodents.

**Methods:**

We assessed whether splenic sheep red blood cell (SRBC) T-dependent antibody response data, measured as antibody-forming/plaque-forming cells (TDAR AFC/PFC), were sufficiently recoverable for structured synthesis and quantitative reanalysis. Recoverability was defined as the availability of endpoint information needed for reuse, including group size, mean, and variance when quantitative reanalysis was attempted.

**Results:**

Fifteen records were screened, and 12 records were retained across vanadium, chromium, tungsten, cadmium, and lead; one of these 12 records contained separate lead and cadmium exposure arms. Three retained records were CEBS package-level studies, yielding 30 module-level entries spanning primary AFC, serum anti-SRBC IgM, natural killer (NK) cell function, splenic phenotype, hematology, body weight, and organ weights. Only one retained study, which examined sodium metavanadate in female B6C3F1/N mice exposed via drinking water for 28 days, provided a fully recoverable primary dataset with extractable group sizes, means, and variances, enabling quantitative reanalysis of five AFC contrasts from 31.3 to 500 ppm. AFC/10^6^ spleen cells shifted from +5.2% at 31.3 ppm to –29.6% at 500 ppm (lnRR –0.351, 95% CI –0.634 to –0.067), and benchmark-like thresholds for 10%, 20%, and 25% AFC decline were estimated at approximately 84, 184, and 283 ppm, respectively. The same package revealed marked assay- and normalization-dependent divergence, with plaque-based AFC declining, ELISpot-based IgM AFC increasing, and serum IgM and NK responses showing limited concordance.

**Discussion:**

Overall, CEBS module-level data supported a stronger endpoint-centered synthesis than narrative review alone. However, incomplete endpoint recoverability, rather than study scarcity, remained the main barrier to broader quantitative integration across oral metal studies.

## Introduction

Heavy-metal immunotoxicity is often discussed as a broad toxicological category, yet the experimental literature is far less uniform than such summaries imply. Across oral rodent studies, investigators have measured humoral function, natural killer (NK) cell activity, splenic immunophenotype, mitogen responsiveness, delayed-type hypersensitivity, host resistance, hematology, and organ-weight changes, often within partially overlapping but biologically non-equivalent study designs (Blakley, 1985; 1988; Borgman et al., 1986; Chowdhury et al., 1987; Frawley et al., 2023; Frawley et al., 2016; Koller et al., 1976; Lawrence, 1981; Mudzinski, 1986; Ohsawa et al., 1988; Shipkowski et al., 2017). This heterogeneity is not merely inconvenient; it limits functional inference. When studies with different routes, assay targets, and immune domains are aggregated without endpoint discipline, the resulting synthesis can overstate coherence and understate mechanistic ambiguity. Accordingly, the overarching goal of this systematic review was to determine how much of the evidence on oral rodent heavy-metal immunotoxicity can be recovered, structured, and quantitatively reanalyzed using Chemical Effects in Biological Systems (CEBS)-based criteria focused on endpoint recoverability. Here, an endpoint refers to a specific immune measurement, and recoverability refers to whether the information needed to reuse that measurement can be obtained.

Within that landscape, splenic sheep red blood cell (SRBC) T-dependent antibody response, measured as antibody-forming/plaque-forming cells (TDAR AFC/PFC), remains the strongest anchor endpoint for cross-study inference. The assay integrates antigen presentation, T-cell help, and B-cell effector function into a single functional readout. It has long been regarded as the conventional humoral immunotoxicity assay in rodents (Ladics, 2007; Ladics, 2018). This endpoint choice is also supported by the foundational National Toxicology Program (NTP)/Luster immunotoxicity testing framework, which identified the splenic antibody PFC response and cell-surface marker analysis as among the most predictive assays for detecting immunotoxicants; combinations of two or three immune parameters achieved greater than 90% concordance in rodent test batteries (Luster, 1988; Luster, 1992; Luster, 1993). This integrative property is precisely what distinguishes it from serum anti-SRBC IgM, NK-cell measures, or descriptive splenic phenotyping. Those endpoints are biologically valuable, but none alone resolves the net functional humoral response. Accordingly, serum anti-SRBC IgM and splenic immunophenotyping were retained as supporting measures of concordance, serving as secondary readouts to assess whether the primary AFC/PFC signal was biologically consistent across related immune domains.

The Chemical Effects in Biological Systems (CEBS) database, maintained by the National Toxicology Program (NTP), is the natural infrastructure for this question because it is a curated and publicly accessible toxicology resource that integrates study design, endpoint modules, and downloadable data across studies (Martini et al., 2022). In this manuscript, CEBS-based refers to an approach in which CEBS was used as the primary source for identifying study packages, extracting structured endpoint information, and assessing whether data could be reused for synthesis or reanalysis. Importantly, CEBS is not merely a publication index. For some compounds, it exposes module-level immunotoxicity datasets that extend beyond the narrative content of the corresponding article and include structured endpoints such as primary AFC, serum anti-SRBC IgM, NK function, splenic phenotyping, hematology, body weight, and organ weights. This curation is scientifically consequential because it organizes experimental metadata and endpoint-level results in reusable formats, supporting independent verification, secondary analysis, and cross-study comparison. That depth creates an opportunity for a more rigorous and transparent evidence synthesis, one that asks not only whether studies exist, but which endpoints are actually recoverable for structured synthesis and reanalysis, and how that recoverability shapes the conclusions that can be drawn. By improving access to structured toxicology data, CEBS can also help the broader scientific community refine evidence standards, identify data gaps, and accelerate cumulative advances in immunotoxicology.

The critical gap is therefore not the lack of oral rodent metal studies. Rather, it is the absence of a CEBS-based framework focused on data recoverability that distinguishes between (i) primary humoral data that are fully recoverable, (ii) records that provide only partial quantitative information, and (iii) records that remain biologically informative but are quantitatively under-delivered. CEBS remains a powerful repository (Martini et al., 2022), but quantitative accessibility varies across compounds and modules, and the distinction between “study present” and “endpoint recoverable” is scientifically consequential. This recoverability gap should not be interpreted simply as a limitation of older studies, many of which predated current expectations for structured endpoint reporting, public-data access, and curation of immunotoxicity data. Thus, the non-uniformity of the dataset was not an incidental weakness, but the condition being evaluated: whether legacy oral metal immunotoxicity studies can still support modern, transparent evidence synthesis when interrogated through a curated toxicology resource. This is especially important for oral heavy-metal immunotoxicity, where chromium(VI), vanadium, tungsten, cadmium, and lead have all been studied, but not all can be interrogated at the same analytical depth (Blakley, 1985; Borgman et al., 1986; Chowdhury et al., 1987; Frawley et al., 2023; Frawley et al., 2016; Koller and Kovacic, 1974; Lawrence, 1981; Mudzinski, 1986; Ohsawa et al., 1988; Shipkowski et al., 2017).

Accordingly, this study was designed as a CEBS-based, endpoint-centered systematic review of oral rodent heavy-metal immunotoxicity, anchored on splenic SRBC-TDAR AFC/PFC. Rather than attempting a broad narrative review or a pooled meta-analysis, we focused on whether endpoint-level evidence could be recovered, structured, and quantitatively reanalyzed. Serum anti-SRBC IgM, NK activity, splenic immunophenotyping, and systemic toxicity measures were retained as concordance layers rather than as substitutes for endpoints. This structure allowed us to distinguish biologically informative legacy evidence from data that are quantitatively reusable for modern evidence synthesis.

## Methods

To avoid ambiguity, evidence was organized into hierarchical analytical levels. A record was a retained scientific article or CEBS-linked source; a study was an individual *in vivo* experiment; a CEBS package-level study was a CEBS-linked package with multiple endpoint modules for the same test article; a module-level entry was one extracted endpoint module; a primary-endpoint entry was one recoverable splenic SRBC-TDAR AFC/PFC result; and a dose contrast was one exposed-versus-control comparison within the fully recoverable sodium metavanadate study. These levels were tracked separately, so counts of records, primary-endpoint entries, module-level entries, secondary-endpoint rows, and dose contrasts were not treated as interchangeable.

### Study design, eligibility criteria, and analytical scope

This study was conducted as a systematic review with structured evidence synthesis and CEBS-native within-study quantitative reanalysis, rather than as a conventional pooled multistudy meta-analysis. Records were eligible if they involved rodents, oral exposure, heavy metals or metalloids, and direct relevance to splenic SRBC-TDAR AFC/PFC. Records were excluded if they were non-rodent, non-oral, centered only on non-comparable immune endpoints, or lacked an *in vivo* humoral response context relevant to SRBC-TDAR.

Secondary endpoints were prespecified as serum anti-SRBC IgM, NK-cell measures, splenic immunophenotyping, and systemic toxicity context, including body weight, spleen weight, organ weights, and selected hematology parameters where available. Records could still be retained for structured synthesis when full AFC summary statistics were unavailable, provided they contributed biologically relevant directional, percent-change, fold change, or statistical information for the primary endpoint. This hierarchy was aligned with the Luster tier-testing literature, which treated T-dependent antibody responses and lymphocyte subset enumeration as complementary, highly informative immune measures rather than interchangeable endpoints.

The design was intentionally restrictive to maximize biological comparability and prevent endpoint drift. These criteria were not intended to demonstrate the full breadth of CEBS, but to test CEBS under a biologically specific, reproducible immunotoxicity question anchored to a functional humoral endpoint. No protocol was prospectively registered; however, eligibility criteria, endpoint hierarchy, evidence-tier definitions, and reanalysis rules were specified before full extraction and synthesis.

### Data sources and record identification

The primary source was CEBS/NTP, supplemented by PubMed, PubMed Central, and backward citation chaining from included studies and relevant references (Martini et al., 2022). The final search update was performed on 30 March 2026. Searches were restricted to English-language records, and no publication-date limits were applied. Additional database searching beyond CEBS/NTP, PubMed, and PubMed Central was not considered necessary because the review question was deliberately narrow and endpoint-centered, focusing specifically on oral rodent heavy-metal studies directly relevant to splenic SRBC-TDAR AFC/PFC, and because CEBS-linked toxicology packages and their associated publications constituted the core source base for recoverability-aware extraction.

Record identification began from CEBS test-article pages, publication pages, and accessible module-linked files for compounds relevant to oral rodent immunotoxicity. Searches combined route terms (“oral,” “drinking water,” “dosed water,” “feed,” “diet,” and “gavage”), endpoint terms (“SRBC,” “sheep red blood cell,” “TDAR,” “AFC,” “PFC,” “plaque-forming cell,” “ELISpot,” “IgM,” “NK,” and “splenic phenotype”), and metal-specific terms (“vanadium,” “sodium metavanadate,” “chromium,” “hexavalent chromium,” “sodium dichromate dihydrate,” “tungsten,” “sodium tungstate dihydrate,” “cadmium,” and “lead”), with rodent terms added where necessary. A representative PubMed/PMC strategy was ((“oral” OR “drinking water” OR “dosed water” OR “feed” OR “diet” OR “gavage”) AND (“SRBC” OR “sheep red blood cell” OR “TDAR” OR “AFC” OR “PFC” OR “plaque-forming cell” OR “ELISpot” OR “IgM” OR “NK” OR “splenic phenotype”) AND (“vanadium” OR “sodium metavanadate” OR “chromium” OR “hexavalent chromium” OR “sodium dichromate dihydrate” OR “tungsten” OR “sodium tungstate dihydrate” OR “cadmium” OR “lead”) AND (“mouse” OR “mice” OR “rat” OR “rats” OR “rodent”)).

Deduplication was performed manually using title, authorship, year, compound, exposure context, and endpoint focus, with CEBS-linked packages and corresponding publications merged before eligibility assessment to avoid duplicate counting of the same evidentiary source. Screening, eligibility assessment, and full extraction were performed by two reviewers, and all retained records were rechecked against the original CEBS-linked or article source during full-text extraction. The final package comprised 15 screened records, of which 12 were retained for structured synthesis. One record was excluded for non-oral exposure, one for lack of direct relevance to splenic SRBC-TDAR AFC/PFC, and one for the absence of a comparable *in vivo* humoral response context.

### Evidence-tier classification, recoverability framework, and CEBS module mining

Included records were assigned to one of three mutually exclusive primary-evidence tiers: 1) fully quantitative primary evidence, defined as recoverable group size, central tendency, and variance for splenic SRBC-TDAR AFC/PFC sufficient for direct effect-size estimation; 2) partial quantitative primary evidence, defined as biologically relevant primary-endpoint data with recoverable percent changes, fold changes, or directional/statistical information but without a full variance structure; and 3) narrative-only primary evidence, defined as biologically relevant primary-endpoint information without sufficiently recoverable numeric structure.

A recoverability matrix was constructed for each included record across primary AFC, serum anti-SRBC IgM, NK, splenic phenotype, body weight, organ weight, hematology, CEBS modularization, and individual-animal data availability. For CEBS-native records, module-level information was extracted directly from accessible test-article pages, linked study summaries, and module files. Module entries were recorded for primary AFC, serum anti-SRBC IgM, NK activity, ELISpot, splenic phenotype, body weight, body weight gain, organ weights, hematology, leukocyte differentials, water consumption, compound consumption, and related cell-mediated endpoints. Three included records were CEBS-native package-level studies: sodium metavanadate, sodium dichromate dihydrate (Cr(VI)), and sodium tungstate dihydrate. Together, these contributed 30 module-level entries: 15 for sodium metavanadate, 7 for chromium(VI), and 8 for sodium tungstate dihydrate.

### Data extraction and harmonization

For each included record, we extracted compound identity, CASRN, parent metal, species, strain, sex, route, duration, dose, dose unit, endpoint family, endpoint metric, recoverability depth, and source mode. For fully quantitative primary data, we additionally extracted the means, standard errors, and group sizes for the control and exposed groups, and derived percent changes. When standard error was reported without standard deviation, SD was derived as SE × √n. Dose was retained on the original study scale, most commonly ppm or mg/L in drinking water, and a natural-log-dose term was derived for within-study trend analysis. Endpoint harmonization was conservative: plaque-based AFC, total AFC/spleen, serum anti-SRBC IgM, ELISpot-based IgM AFC, NK activity, and splenic cell subsets were retained as analytically distinct endpoints rather than pooled into a single immune score.

### Quantitative reanalysis

The prespecified primary effect size for the fully quantitative AFC dataset was the log response ratio (lnRR), defined as ln (mean exposed/mean control). Because only one independent study provided a fully recoverable variance structure, a pooled multistudy random-effects meta-analysis was not estimable. Accordingly, quantitative reanalysis was restricted to the contrast level within the sodium metavanadate study and was not treated as a cross-metal quantitative analysis.

A descriptive inverse-variance-weighted within-study linear fit was estimated across the five sodium metavanadate lnRR contrasts on the ln-dose scale to summarize the internal dose pattern. This was explicitly treated as a within-study descriptive model, not as a pooled meta-regression. To extend the reanalysis beyond the primary AFC readout, all recoverable module-level quantitative endpoints for sodium metavanadate were extracted into a multimodule quantitative dataset, including plaque-based AFC/10^6^ spleen cells, AFC/spleen, spleen cellularity, spleen weight, serum anti-SRBC IgM, ELISpot-based IgM AFC, NK activity, splenic B-cell and NK-cell percentages, absolute B-cell and NK-cell counts, total T-cell:B-cell ratio, and terminal body weight.

Using the within-study AFC dose–response pattern, descriptive benchmark-like thresholds for 10%, 20%, and 25% decline in AFC/10^6^ spleen cells were estimated by interval-based log-dose interpolation and by the fitted inverse-variance-weighted within-study lnRR model. These thresholds were reported as descriptive benchmark-like metrics rather than as formal regulatory benchmark dose/benchmark dose lower limit (BMD/BMDL) estimates.

### Secondary-endpoint synthesis

Secondary endpoints were analyzed as concordance or discordance layers relative to the primary AFC signal. Serum anti-SRBC IgM, NK-cell function, NK-cell fraction, splenic phenotype, body weight, spleen weight, hematology, and related immune context were summarized both within the multimodule quantitative reanalysis for sodium metavanadate and across the broader cross-metal package. Secondary endpoints were never treated as substitutes for the primary AFC endpoint.

### Risk-of-bias and reporting considerations

Many included records predated current animal-reporting standards, and for several older cadmium and lead studies, only abstract-level or partially reported numerical data were recoverable. Formal study-level risk-of-bias coding was therefore constrained by incomplete reporting of randomization, blinding, allocation procedures, and attrition (Hooijmans et al., 2014; Page et al., 2021; Percie Du Sert et al., 2020). These limitations were treated primarily as reporting and recoverability constraints rather than as evidence of systematic invalidity. The manuscript structure follows PRISMA principles (Page et al., 2021), and interpretive caution was guided by ARRIVE 2.0 and the SYRCLE framework (Hooijmans et al., 2014; Percie Du Sert et al., 2020).

## Results

### Study selection and CEBS-native evidence architecture

**FIGURE 1:**
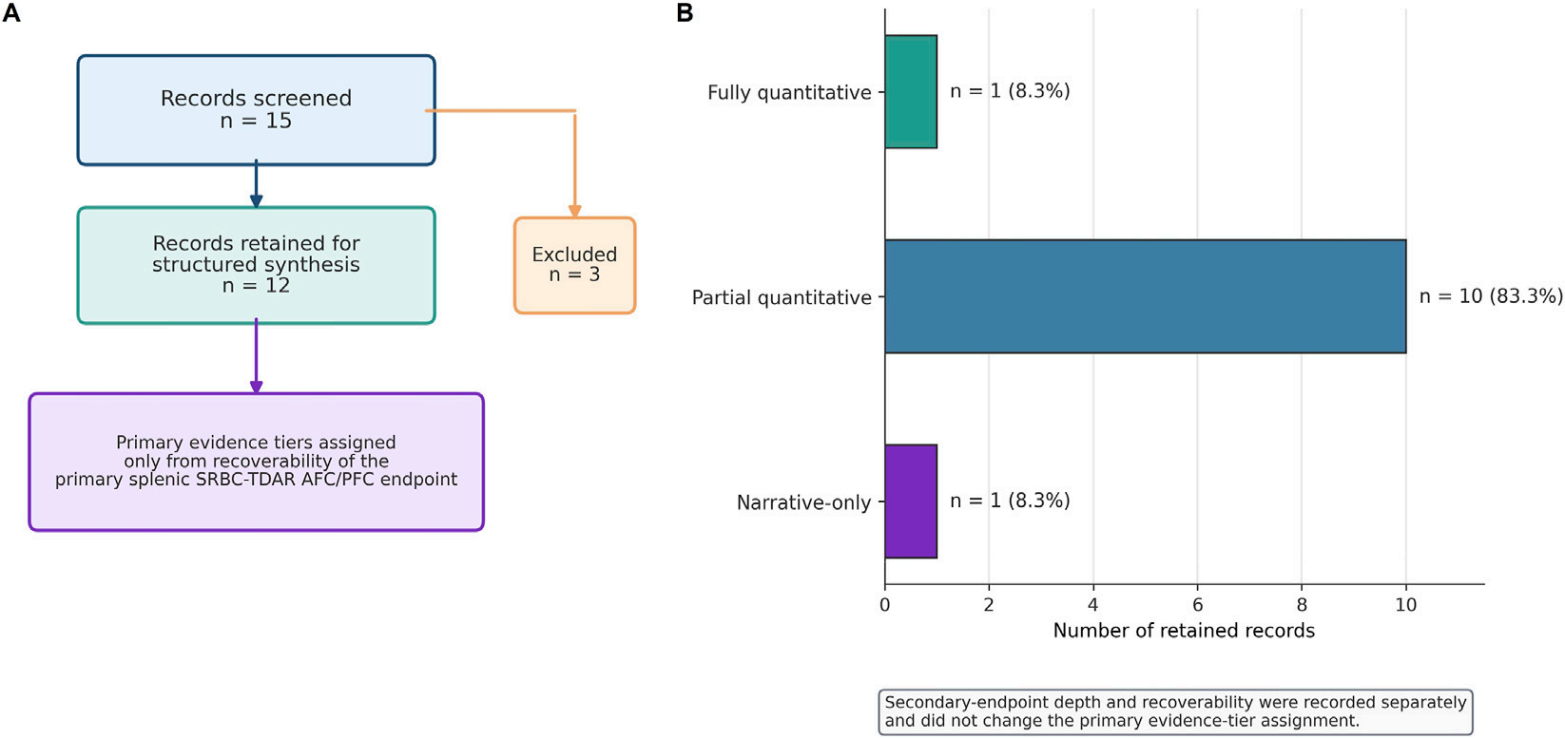
Study selection and mutually exclusive primary evidence-tier classification. **(A)** Screening workflow: 15 screened records, 12 retained for structured synthesis, and 3 excluded. Exclusions were due to non-oral exposure (n = 1), lack of direct relevance to splenic SRBC-TDAR AFC/PFC (n = 1), or absence of a comparable *in vivo* humoral response context (n = 1). **(B)** Mutually exclusive primary evidence-tier distribution among retained records, assigned solely on the basis of the recoverability of the primary splenic SRBC-TDAR AFC/PFC endpoint: fully quantitative (n = 1, 8.3%), partial quantitative (n = 10, 83.3%), and narrative-only (n = 1, 8.3%). Secondary-endpoint depth and recoverability were recorded separately and did not affect primary evidence-tier assignment.

Of the 15 screened records, 12 were retained for structured synthesis across 5 principal metals (vanadium, chromium, tungsten, cadmium, and lead), with 1 additional oral study containing separate lead and cadmium exposure arms, retained because it provided primary humoral information. Among the included records, 1 was fully quantitative, 10 were partially quantitative, and 1 was narrative-only. This architecture was not incidental; it was the defining empirical feature of the dataset. The distribution of evidence tiers and endpoint recoverability, which defines the dataset’s analytical constraints, is summarized in Figure 1.

The recoverability matrix showed that primary AFC was fully recoverable in only 1 of 12 included records, whereas the remaining records were either partial quantitative or narrative-only for the primary endpoint. Secondary-endpoint recoverability was asymmetrical rather than uniformly poor. Full summary statistics were recoverable for serum anti-SRBC IgM in 2 records, for NK function in 1 record, and for splenic phenotype in 1 record. Three included compounds, which were sodium metavanadate, chromium(VI), and sodium tungstate dihydrate, had CEBS-native package-level depth. Still, only sodium metavanadate exposed a fully modularized immunotoxicity package with accessible summary statistics across multiple immune layers. Thus, the main analytical bottleneck was not the absence of oral rodent metal studies, but the uneven public recoverability of the primary endpoint. Table 1 summarizes the retained evidence base and shows that most oral rodent heavy-metal studies are only partially recoverable for the primary AFC/PFC endpoint. The main conclusion from this distribution is that endpoint recoverability, rather than simple study count, is the central barrier to pooled cross-metal synthesis.

**TABLE 1:** Characteristics of included rodent oral heavy-metal studies evaluating splenic SRBC-TDAR AFC/PFC, with mutually exclusive primary-evidence tiers.

Study	Metal/Compound	Model/Route/Exposure	Primary-endpoint availability	Primary-evidence tier	Secondary-endpoint depth/Recoverability notes	References
1	Sodium metavanadate	Female B6C3F1/N mouse; drinking water; 28 days; 31.3–500 ppm	Full AFC/10^6^ spleen cells dataset with recoverable mean, SE, and n	Fully quantitative	CEBS package-level study with multimodule summary statistics	Frawley et al. (2023)
2	Chromium(VI)/sodium dichromate dihydrate	Oral drinking-water study in rats and mice; multiple doses	AFC/PFC reported with directional and quantitative information but without fully recoverable variance across contrasts	Partial quantitative	CEBS-linked record; secondary endpoints partly recoverable	Shipkowski et al. (2017)
3	Sodium tungstate dihydrate	Female B6C3F1/N mouse; oral drinking water; multiple doses	Primary AFC/PFC endpoint relevant but not recoverable as a variance-supported quantitative dataset	Narrative-only	Secondary endpoints were informative, but the primary humoral signal remained at a narrative level	Frawley et al. (2016)
4	Cadmium chloride	Mouse; oral exposure	Suppressive AFC/PFC information is recoverable directionally/quantitatively but no full variance structure	Partial quantitative	Primary humoral perturbation reported	Blakley (1985)
5	Cadmium	Mouse; oral exposure	Suppression after SRBC priming was reported without full recoverable summary statistics	Partial quantitative	Useful for direction and significance only	Ohsawa et al. (1988)
6	Cadmium	Aged mouse; oral exposure	Humoral endpoint reported without a full recoverable variance structure	Partial quantitative	Supports broader cadmium signal but not direct effect-size estimation	Blakley (1988)
7	Cadmium	Mouse; chronic oral exposure	Time-dependent suppression reported without full variance recovery	Partial quantitative	Partial quantitative primary evidence	Borgman et al. (1986)
8	Cadmium (zinc-modifiable design)	Mouse; oral exposure	Opposite-direction enhancement under zinc-modifiable design; no full variance recovery	Partial quantitative	Informative for direction and biological context	Chowdhury et al. (1987)
9	Lead	Mouse; oral exposure	Fivefold decrease in 19S/IgM AFC/PFC convertible to fold-change evidence but not a full variance structure	Partial quantitative	Strong suppressive signal, partial quantitative only	Koller and Kovacic (1974)
10	Lead	Mouse; oral exposure	*In vivo* plaque-forming-cell response described without a fully recoverable quantitative dataset	Partial quantitative	Primary endpoint relevant but incomplete numerically	Lawrence (1981)
11	Lead	Mouse; oral exposure	Strain-dependent PFC/AFC changes reported without full variance recovery	Partial quantitative	Heterogeneous lead pattern	Mudzinski (1986)
12	Separate lead and cadmium single-exposure arms	Mouse; oral exposure	Primary humoral response reported without full variance recovery	Partial quantitative	Retained for primary humoral relevance in the lead/cadmium evidence base	Koller et al. (1976)

### CEBS module mining reveals asymmetrical endpoint depth across compounds

The module registry contained 30 module-level entries across the 3 CEBS-native compounds. Sodium metavanadate contributed 15 module entries: body weight, body weight gain, water consumption, compound consumption, leukocyte differential, hematology, splenic phenotype, primary AFC, serum anti-SRBC IgM, anti-KLH antibodies, anti-CD3 responses, cytotoxic T-cell activity, NK function, TDAR ELISpot, and organ weights. Chromium(VI) contributed 7 module-level or article-level endpoint entries: specific and total AFC, serum anti-SRBC IgM, NK activity, splenic phenotype, body weight, and red blood cell parameters. Sodium tungstate dihydrate contributed 8 entries: AFC, serum IgM, NK activity, splenic phenotype, cell-mediated immunity, body/systemic context, organ weights, and hematology.

This module asymmetry had direct analytical consequences. Only sodium metavanadate yielded a multimodule quantitative matrix with sufficiently dense endpoint coverage for integrated analysis. Chromium(VI) and sodium tungstate dihydrate remained CEBS-linked but only partially recoverable at the immunotoxicity-module level. The CEBS-native strategy, therefore, identified not only which compounds had relevant studies but also which compounds could actually support analysis beyond the narrative level.

### Quantitative primary reanalysis of sodium metavanadate

The only fully recoverable primary dataset came from sodium metavanadate in female B6C3F1/N mice exposed to dosed water for 28 days. The primary AFC dataset comprised five dose contrasts at 31.3 ppm, 62.5 ppm, 125 ppm, 250 ppm, and 500 ppm against a shared vehicle-control group. The control mean for AFC/10^6^ spleen cells was 1099.7 (n = 7), and each exposed group contained eight animals.

**FIGURE 2:**
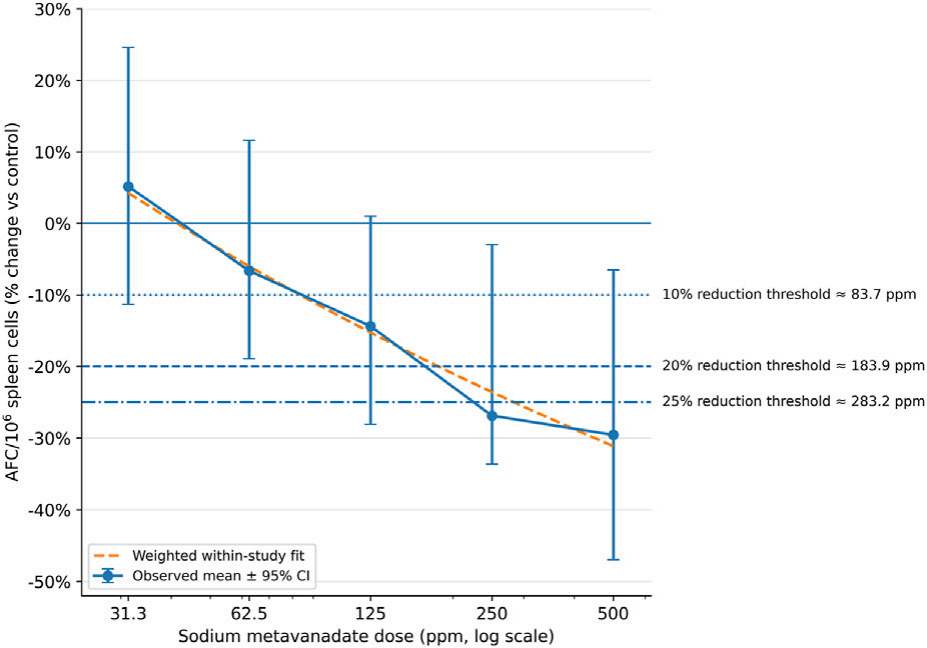
Dose–response relationship for plaque-based AFC/10^6^ spleen cells in female B6C3F1/N mice exposed to sodium metavanadate in drinking water for 28 days. Points represent the mean percent change relative to control for AFC/10^6^ spleen cells at 31.3 ppm, 62.5 ppm, 125 ppm, 250 ppm, and 500 ppm sodium metavanadate; error bars indicate 95% confidence intervals. The dashed curve shows the inverse-variance-weighted within-study fit on the log-dose scale. Horizontal reference lines indicate approximate benchmark-like thresholds for 10%, 20%, and 25% AFC decline (≈83.7 ppm, 183.9 ppm, and 283.2 ppm, respectively). The figure summarizes a progressive within-study suppression of plaque-based AFC/10^6^ spleen cells, with the steepest decline occurring between 125 ppm and 250 ppm. Threshold values are descriptive benchmark-like estimates and should not be interpreted as formal regulatory BMD/BMDL values.

The AFC/10^6^ spleen cells changed from +5.165% at 31.3 ppm to −6.620% at 62.5 ppm, −14.368% at 125 ppm, −26.907% at 250 ppm, and −29.572% at 500 ppm. The strongest effect occurred at 500 ppm (lnRR −0.350575, 95% CI −0.633887 to −0.067263). The absolute mean difference relative to control shifted from +56.8 AFC/10^6^ spleen cells at 31.3 ppm to −72.8, −158.0, −295.9, and −325.2 at 62.5 ppm, 125 ppm, 250 ppm, and 500 ppm, respectively. The steepest downward transition occurred between 125 ppm and 250 ppm, while the transition from 250 ppm to 500 ppm was shallower, indicating attenuation of slope at the upper end. The within-study dose–response pattern for AFC/10^6^ spleen cells, including the progressive decline across the dose range and the approximate benchmark-like thresholds for functional suppression, is illustrated in Figure 2.

The inverse-variance-weighted within-study lnRR fit yielded a slope of −0.149522 lnRR units per ln-dose unit. On that basis, the estimated AFC decline per doubling of dose was approximately 9.8%. Descriptive benchmark-like thresholds for AFC decline were then estimated. For a 10% decline, the weighted-fit threshold was 83.652 ppm, and the interval-interpolated threshold was 84.567 ppm. For a 20% decline, the corresponding values were 183.901 ppm and 170.655 ppm. For a 25% decline, they were 283.165 ppm and 224.987 ppm, respectively. These values should be understood as within-study descriptive thresholds rather than formal benchmark doses, but they provide a more analytically meaningful characterization of the dataset than a simple listing of contrasts. Table 2 compiles the only fully recoverable primary AFC/PFC dataset. It aligns the tabular presentation with the ln-dose scale used for the within-study reanalysis, showing a transition from no consistent effect at 31.3 ppm to marked suppression at 500 ppm.

**TABLE 2:** Fully recoverable primary dataset and within-study effect-size summary for plaque-based splenic SRBC-TDAR AFC/PFC after oral sodium metavanadate exposure in female B6C3F1/N mice.

Dose (ppm)	ln (dose)	AFC/10^6^ spleen cells (% change vs. control)	lnRR	95% CI (lnRR)
31.3	3.4436	5.17%	0.0504	−0.120 to 0.220
62.5	4.1352	−6.620%	−0.0685	−0.210 to 0.110
125	4.8283	−14.368%	−0.1551	−0.330 to 0.010
250	5.5215	−26.907%	−0.3134	−0.410 to −0.030
500	6.2146	−29.572%	−0.3506	−0.634 to −0.067

Control mean = 1,099.7 AFC/10^6^ spleen cells (n = 7). Each exposed group contained eight animals. The original dispersion in the source dataset was reported as standard error (SE); SD was derived as SE × √n as needed for effect-size estimation. The within-study fitted trend shown in the manuscript was estimated on the ln-dose scale; therefore, ln(dose) is shown here instead of log_10_ (dose). Dose contrasts share a common vehicle-control group.

### Multimodule analysis shows assay- and normalization-dependent divergence

**FIGURE 3:**
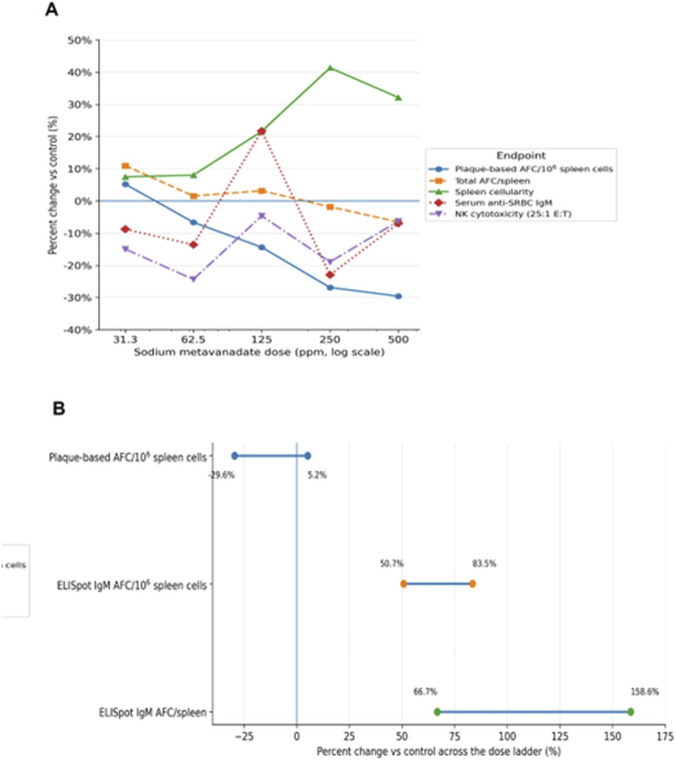
Assay- and endpoint-dependent response patterns following 28-day oral sodium metavanadate exposure in female B6C3F1/N mice. **(A)** Percent change relative to control across sodium metavanadate doses of 31.3, 62.5, 125, 250, and 500 ppm for plaque-based AFC/10^6^ spleen cells, total AFC/spleen, spleen cellularity, serum anti-SRBC IgM, and NK-cell cytotoxicity (25:1 E:T). **(B)** Range of percent changes relative to control across the dose ladder for plaque-based AFC/10^6^ spleen cells, ELISpot IgM AFC/10^6^ spleen cells, and ELISpot IgM AFC/spleen, illustrating assay- and normalization-dependent divergence. The strongest plaque-based AFC response occurred at 500 ppm (lnRR –0.351, 95% CI –0.634 to –0.067), corresponding to a –29.6% change relative to control. AFC, antibody-forming cells; CI, confidence interval; E:T, effector-to-target ratio; IgM, immunoglobulin M; lnRR, log response ratio; NK, natural killer; SRBC, sheep red blood cells.

The divergent dose-resolved patterns across selected non-ELISpot endpoints are shown in Figure 3A, whereas the assay-level divergence between plaque-based AFC and ELISpot-based IgM AFC readouts is summarized in Figure 3B. This distinction is important because the ELISpot-based response moves in the opposite direction to plaque-based AFC suppression and therefore represents one of the most informative sources of discordance in the sodium metavanadate package.

First, the direction of the plaque-based AFC signal depended strongly on the normalization method. While AFC/10^6^ spleen cells declined progressively and reached −29.572% at 500 ppm, total AFC/spleen remained positive or near neutral through 125 ppm (+10.974%, +1.521%, +3.151%) and only turned modestly negative at higher doses (−1.861% at 250 ppm; −6.473% at 500 ppm). This divergence coincided with increased splenic cellularity, which increased by +7.477%, +8.010%, +21.617%, +41.343%, and +32.127% across the five doses. Thus, specific plaque-forming activity per splenocyte declined, while the total cell number increased sufficiently to buffer the total spleen output over most of the dose range.

Second, the ELISpot-based TDAR sensitivity endpoint moved in the opposite direction. ELISpot IgM AFC/10^6^ spleen cells increased +50.741% to +83.523%, and ELISpot IgM AFC/spleen increased +66.730% to +158.619% across the same dose ladder. This divergence between plaque-based AFC suppression and ELISpot-based IgM AFC enhancement is one of the most informative findings in the dataset. It indicates that the same biological package can support very different conclusions depending on the functional readout and normalization framework used. In other words, “TDAR” is not analytically interchangeable across platforms.

Third, secondary endpoints were only partially concordant. Serum anti-SRBC IgM was non-monotonic, shifting −8.743%, −13.591%, +21.738%, −22.949%, and −6.967% across the dose ladder. NK cytotoxicity at a representative 25:1 effector:target ratio changed by −14.964%, −24.369%, −4.617%, −18.940%, and −6.285%, which was directionally suppressive but not dose-ordered in the same way as AFC/10^6^ spleen cells. Splenic percent B cells rose progressively to +12.407% at 500 ppm, percent NK cells declined −10.578% to −19.492%, absolute B cells increased at higher doses (+13.093% and +11.093% at 250 ppm and 500 ppm), and absolute NK cells were consistently reduced (−13.873% to −21.215%). Terminal body weight showed only modest downward shifts (0.0% to −4.527%), whereas spleen weight increased +3.692% to +11.897%. Overall, these secondary endpoints showed partial concordance with AFC/10^6^ spleen cells and provided quantitative context for the primary endpoint.

### Cross-metal primary-endpoint landscape

The primary cross-metal dataset contained 23 primary-endpoint entries, of which 5 were fully quantitative, and 18 were partially quantitative. Across the primary entries that could be expressed numerically as percent change versus control, vanadium spanned +5.2% to −29.6%, chromium(VI) spanned +28.6% to +66.0%, cadmium contributed a directly reported suppressive estimate of −28.2%, and lead spanned −80.0% to +17.0%.

For chromium(VI), the primary-endpoint pattern was numerically substantial but internally unstable. Specific AFC/10^6^ spleen cells increased +66.0% in F344/N rats at 57.3 ppm and +28.6% and +31.7% in B6C3F1 mice at 31.3 ppm and 62.5 ppm, while total AFC/spleen increased +62.0% in rats and +34.0% in mice. However, the record explicitly described these changes as sporadic, non-dose-dependent, and non-reproducible. Chromium(VI), therefore, contributed a signal without coherence.

For sodium tungstate dihydrate, the primary humoral endpoint remained narrative-only and largely negative. The accessible package supported no convincing AFC suppression signal and no parallel decrease in serum anti-SRBC IgM or NK activity, although the study did indicate changes in cell-mediated and hematologic context at higher doses. Tungstate thus served as a negative humoral comparator.

For cadmium, the structured synthesis yielded the clearest recurrent oral humoral disruption pattern outside vanadium. The strongest directly quantifiable cadmium signal was the −28.2% suppression reported by Blakley at the highest exposure. Ohsawa et al. (1988) showed significant suppression after SRBC priming at 300 ppm, Borgman et al. (1986) showed time-dependent suppression at 5 days but not 7 days, and Chowdhury et al. (1987) showed an opposite-direction enhancement under a zinc-modifiable design. Cadmium, therefore, did not behave as a uniform suppressor, but it repeatedly perturbed oral humoral function across study designs.

For lead, the record set remained markedly heterogeneous. Koller et al. (1976) supported strong suppression (−80.0%, derived from a reported fivefold decrease in 19S/IgM AFC), Lawrence (1981) retained a qualitative null *in vivo* plaque-forming cell response, and Mudzinski (1986) showed +17.0% in BALB/c-specific PFC/10^6^ splenocytes without a parallel change in total PFC/spleen. The single-dose study, with separate lead and cadmium exposure arms, was further interpreted by immunoglobulin class, with lead associated with increased IgM AFC on Day 5 but decreased IgG AFC on Day 2. Lead was therefore not directionally stable within the current package.

### Secondary-endpoint concordance across metals

The secondary cross-metal dataset contained 71 rows spanning serum anti-SRBC IgM, natural killer cells, splenic immunophenotype, systemic context, hematology, T-cell and B-cell proliferation, delayed-type hypersensitivity, autoimmunity context, mitogen response, serum antibodies, renal immune pathology, host resistance, and route dependence. The most recoverable and interpretable secondary layers were serum IgM and splenic phenotype. Table 3 summarizes the secondary immune endpoints evaluated alongside AFC/PFC and shows that these measures primarily serve as concordance layers: they contextualize the primary signal but do not replace AFC/PFC as the principal synthesis anchor.

**TABLE 3:** Secondary immune endpoints in rodent oral heavy-metal studies were evaluated alongside splenic SRBC-TDAR AFC/PFC.

Metal/Compound	Species	Endpoint	Direction of effect	Quantitative availability	Interpretation relative to AFC/PFC
Sodium metavanadate	Mouse (B6C3F1/N)	Serum anti-SRBC IgM	Non-monotonic	Fully quantitative within package	Discordant with plaque-based AFC suppression
Sodium metavanadate	Mouse (B6C3F1/N)	NK-cell lytic activity	Weakly suppressive/inconsistent	Fully quantitative within package	Does not mirror the AFC dose pattern
Sodium metavanadate	Mouse (B6C3F1/N)	Splenic NK cell %	Dose-related decrease	Fully quantitative within package	Cellular redistribution accompanies AFC decline
Sodium metavanadate	Mouse (B6C3F1/N)	Splenic B cell %	Increase with dose	Fully quantitative within package	Suggests compensatory or remodeling response
Chromium(VI)	Mouse/rat	Serum anti-SRBC IgM	Mild increase/inconsistent	Partial quantitative	Does not support a reproducible AFC signal
Chromium(VI)	Mouse/rat	Splenic immunophenotyping	Variable	Partial quantitative	No stable concordance with AFC
Sodium tungstate dihydrate	Mouse	Serum anti-SRBC IgM	No effect	Partial quantitative	Concordant with the lack of AFC suppression
Sodium tungstate dihydrate	Mouse	NK activity	No effect	Partial quantitative	No functional NK evidence of immunotoxicity
Cadmium (various salts)	Mouse/rat	Serum anti-SRBC IgM	Decrease or dysregulation	Partial quantitative	Supports recurrent humoral perturbation
Cadmium (various salts)	Mouse/rat	Splenic phenotype/proliferation	Altered lymphocyte populations	Partial quantitative	Consistent with broader immune disruption
Lead (various compounds)	Mouse/rat	Serum anti-SRBC IgM	Mixed/dose-dependent	Partial quantitative	Inconsistent with the primary AFC direction
Lead (various compounds)	Mouse/rat	NK activity/host resistance	Variable	Narrative/partial	No stable secondary concordance
Lead or cadmium single-dose study (separate arms)	Mouse	Serum IgM/phenotype	Dysregulated	Narrative	Suggests interaction but not yet quantifiable

In chromium(VI)-treated mice, serum IgM titers were only modestly increased (+1.922% to +11.963%) across the mouse dose ladder and did not track the magnitude or instability of AFC changes. Splenic B-cell percentages changed only slightly (−2.584% to +6.362%), NK-cell percentages remained near baseline in most groups, and absolute macrophages decreased in mice by −4.545% to −33.333%. Body weight gain and erythroid parameters showed limited changes at higher doses. This pattern again supports the conclusion that the AFC increases were neither broad nor robustly integrated across immune layers.

In sodium tungstate dihydrate, the secondary pattern was broadly concordant with the null humoral signal. The accessible package showed no effect on serum anti-SRBC IgM, no effect on NK activity, and no meaningful splenic phenotype changes, although neutrophils declined by 17%–33%, lymphocytes rose by 14% at 250–1,000 mg/L, and monocytes fell by 15%–21% at 500–1,000 mg/L. Thus, tungstate appeared largely humoral-negative but not globally inert.

Across cadmium and lead, secondary endpoints reinforced heterogeneity rather than resolving it. Cadmium included records with preserved ConA responses, enhanced LPS-driven B-cell proliferation, absent delayed-type hypersensitivity, and autoimmunity-related findings. Lead included null serum antibody readouts in some settings, altered host-resistance context, and study-specific immune effects without a stable secondary signature. These patterns confirm that serum IgM, NK, and splenic phenotype are useful as context but do not replace AFC/PFC as the primary anchor for synthesis. Table 4 integrates mutually exclusive metal-level primary-evidence tiers and cross-metal interpretations, showing that vanadium provides the most robust recoverable oral TDAR suppression signal; cadmium shows recurrent but only partially recoverable humoral disruption; and chromium(VI), tungstate, and lead do not yield a single stable, reproducible pattern.

**TABLE 4:** Mutually exclusive metal-level primary-evidence tiers and primary-endpoint interpretation across oral heavy metals in rodents.

Metal	Primary-evidence tier	AFC/PFC direction	Consistency	Secondary-endpoint concordance	Overall interpretation
Vanadium (sodium metavanadate)	Fully quantitative	Dose-dependent suppression	High within the study	IgM non-monotonic; NK weakly inconsistent; splenic NK ↓, B cells ↑	Most robust recoverable evidence of functional humoral suppression
Chromium(VI)	Partial quantitative	Sporadic increases	Low (non-dose-dependent, non-reproducible)	IgM and phenotype inconsistent	No reproducible effect on oral TDAR AFC/PFC
Tungsten (sodium tungstate dihydrate)	Narrative only	No consistent effect	Moderate for a negative signal	IgM and NK largely negative	No evidence of TDAR suppression under tested conditions
Cadmium (various salts)	Partial quantitative	Suppression or dysregulation	Moderate (recurrent across studies)	IgM decrease/dysregulation; altered splenic phenotype	Recurrent indication of oral humoral immune disruption
Lead (various compounds)	Partial quantitative	Mixed/heterogeneous	Low (design-dependent)	IgM and NK variable	No single stable pattern; context-dependent effects

## Discussion

Our work shows that the strongest insight available from public oral rodent metal data does not come from asking whether “heavy metals are immunotoxic” in general, but from asking which endpoint is actually recoverable, which signal is functionally coherent, and how far that coherence extends across related immune layers. Under that framing, splenic SRBC-TDAR AFC/PFC remained the most defensible primary endpoint, and endpoint recoverability emerged as the key determinant of analytical strength.

A key strength of this study is that it does not rely solely on a single AFC contrast table but instead integrates recoverability-aware evidence synthesis with within-study quantitative reanalysis where the data permit it. By mining CEBS at the module level, the study recovers a much richer quantitative structure for sodium metavanadate and a more explicit cross-metal architecture for chromium(VI), tungsten, cadmium, and lead. The result is not a pooled multimetal estimate, which the package still does not support, but a stronger evidence synthesis that combines biological interpretation with a methodological demonstration of what CEBS can and cannot currently support.

The sodium metavanadate package is the clearest example. If interpreted solely on the basis of plaque-based AFC/10^6^ spleen cells, the study appears to show straightforward dose-related humoral suppression. If interpreted solely from the total AFC/spleen, the same study appears much weaker, with only a mild negative effect at high doses. If interpreted through ELISpot, the direction reverses entirely. If interpreted through serum anti-SRBC IgM, the pattern becomes non-monotonic. If interpreted in terms of NK lytic function, the changes are shallow and irregular. That is precisely why this package is so valuable: it demonstrates, within a single CEBS-native study, that humoral immunotoxicity depends strongly on both the chosen endpoint and its normalization. The most defensible choice remains the plaque-based SRBC-TDAR AFC/PFC assay, as it is the historical anchor assay (Ladics, 2007; Ladics, 2018). However, this study also shows that even among TDAR-related measurements, assay-dependent divergence can be substantial. This interpretation is consistent with the earlier Luster studies, which identified the PFC response and lymphocyte/cell-surface marker analysis as highly sensitive components of immunotoxicity screening. Thus, our contribution is not to newly establish PFC/AFC as the preferred endpoint, but to show how this established endpoint priority performs in recoverability-aware synthesis and CEBS-based reanalysis of oral metal immunotoxicity data.

The difference between AFC/10^6^ spleen cells and AFC/spleen is especially instructive. Because spleen cellularity and spleen weight expanded while specific AFC activity declined, total AFC/spleen was partially buffered. This suggests that the dominant biological phenomenon is not a simple collapse of total humoral capacity but rather a change in the efficiency or composition of the splenocyte compartment’s response. That interpretation remains inferential rather than mechanistic proof, but it is strongly supported by the simultaneous increases in spleen cells and B-cell percentage, the reduction in the NK-cell fraction, and only a mild reduction in body weight. The data are consistent with selective remodeling of splenic immune architecture in response to vanadate exposure.

The benchmark-like AFC thresholds strengthen that interpretation by moving the within-study quantitative reanalysis from descriptive contrast listing to threshold-oriented toxicology. Even without formal BMD software and without multiple independent studies, the within-study data support a coherent dose region for the onset of measurable functional impairment. The convergence of the 10% decline threshold at approximately 84 ppm across both interpolation and weighted-fit approaches is particularly encouraging. Divergence widens at the 20%–25% range, as expected in a sparse five-dose design, but the threshold estimates remain useful as descriptive anchors. That adds a level of toxicological maturity that a narrative summary alone would not provide.

These findings also have potential relevance to human health risk assessment, particularly in hazard identification and evidence prioritization. Oral exposure is a key environmental route for heavy metals through drinking water, diet, and contaminated media, so rodent oral immunotoxicity data can help identify immune endpoints that may warrant consideration in environmental health evaluations. However, the sodium metavanadate threshold estimates reported here are mouse drinking-water, within-study descriptive values, not human-equivalent doses or regulatory points of departure. Their main value in risk assessment is to indicate which functional humoral endpoints are sufficiently recoverable to support future dose–response modeling, cross-study comparison, and more formal translation when comparable datasets and toxicokinetic information become available.

Chromium(VI) and sodium tungstate dihydrate serve an important interpretive role within this evidence structure. Chromium(VI) is not simply a partial quantitative nuisance; rather, it provides a cautionary example of why effect magnitude without dose structure or reproducibility is insufficient for strong inference. Tungstate serves as the complementary case: it is a humoral-negative record with limited secondary perturbation, helping define the lower boundary of the response space. Together, they sharpen specificity rather than dilute it. A field in which every metal appears suppressive under every endpoint would be easier to summarize but much less credible.

Cadmium and lead also become more interpretable under this framework. Cadmium stands out not simply because it “suppresses immunity,” but because it shows recurrent perturbation of humoral function across designs, including suppressive and dysregulatory modes. Lead, by contrast, remains heterogeneous even after the evidence is tiered and endpoint-centered. That difference matters. It suggests that the field should stop treating “metals” as a single analytic category and instead distinguish metals that produce recurrent humoral perturbation from metals whose public record is internally unstable or context-dependent.

The review also reveals something of broader methodological value: the limiting variable is not the existence of a study but the recoverability of the endpoint. CEBS is indeed the correct database for this problem (Martini et al., 2022). It contains the right compounds, study packages, and endpoint logic. However, public extraction remains uneven. Sodium metavanadate is a proof-of-principle case showing what CEBS can support when modules are accessible. Chromium(VI) and sodium tungstate dihydrate show the next layer down: biologically relevant, CEBS-linked, but only partly recoverable. The older cadmium and lead records constitute the legacy literature layer: valuable but variably quantified and only partly compatible with modern structured reanalysis. That recoverability gradient is itself one of this article’s most important contributions. A more contemporary and uniform dataset would be expected to contain harmonized endpoint definitions, complete group-level means, variance estimates, and sample sizes, comparable dose metrics, and accessible module-level results across compounds. Such a dataset could support formal cross-study meta-analysis and dose–response modeling. In contrast, the present legacy dataset provides a realistic test case for defining the current limits of CEBS-based data recoverability and identifying the specific data elements needed for future immunotoxicity synthesis.

This study has several clear strengths. It is endpoint-disciplined, route-restricted, and explicitly recoverability-aware. It treats CEBS as an analytical resource rather than as a simple literature index. It uses a within-study quantitative reanalysis across multiple modules to extract more signals from the best available study. It also avoids a common failure mode in evidence synthesis by not implying that a pooled multimetal estimate exists when the package does not support it. The main limitations are equally clear. Only one independent study yielded a fully recoverable primary AFC dataset; the benchmark-like thresholds are descriptive rather than formal regulatory estimates; many older studies are incompletely reported; and the package does not yet support pooled subgroup analysis, formal between-study heterogeneity modeling, or publication-bias assessment (Hedges et al., 2010; Hooijmans et al., 2014; Page et al., 2021; Percie Du Sert et al., 2020; Tipton, 2015; Viechtbauer, 2010).

What would materially change the inference is specific and tractable: broader public recovery of AFC tables reporting group means, variance estimates, and sample sizes, especially for chromium(VI), tungsten, cadmium, and lead records already shown here to be endpoint relevant. If those modules or tables became recoverable, the field could shift from a CEBS-native structured evidence synthesis anchored in a single within-study multimodule quantitative reanalysis toward a true multistudy quantitative framework. Until then, the most defensible position is to treat endpoint recoverability as a scientific variable in its own right.

## Conclusion

A CEBS-native, recoverability-aware strategy yields a stronger and more informative immunotoxicology article than a broad narrative review of oral heavy metals. In the current public-data environment, the strongest inference is not a pooled cross-metal estimate, but a metal-specific, endpoint-centered systematic review with structured evidence synthesis and within-study quantitative reanalysis anchored on splenic SRBC-TDAR AFC/PFC. Sodium metavanadate provides the only fully recoverable primary dataset. It shows a dose-ordered decline in plaque-based AFC/10^6^ spleen cells together with strong multimodule discordance across ELISpot, serum IgM, NK, splenic phenotype, and tissue context. Cadmium shows the clearest recurrent pattern of oral humoral perturbation across the broader literature, whereas chromium(VI), sodium tungstate dihydrate, and lead define the limits of generalization through non-reproducibility, humoral negativity, or marked heterogeneity. The principal barrier to stronger quantitative synthesis is therefore not conceptual but infrastructural: recoverable AFC tables reporting group means, variance estimates, and sample sizes for additional oral metal studies. Until such datasets become more broadly recoverable, the most robust quantitative inference will remain within-study rather than cross-metal. Therefore, the direct scientific relevance of this work lies in defining the boundary between biologically informative legacy evidence and publicly available, quantitatively reusable toxicology data.

## Data Availability

The original contributions presented in the study are included in the article/Supplementary Material, further inquiries can be directed to the corresponding author.
